# From data to meaning: nursing clinical judgement in the age of artificial intelligence

**DOI:** 10.1177/17449871251386307

**Published:** 2025-11-10

**Authors:** José Alves, Paulo Alves

**Affiliations:** Ph.D. Candidate, Center for Interdisciplinary Research in Health, Faculty of Health Sciences and Nursing, Catholic University of Portugal, Porto, Portugal; Associate Professor, Center for Interdisciplinary Research in Health, Faculty of Health Sciences and Nursing, Catholic University of Portugal, Porto, Portugal

## Introduction

We stand at a critical juncture in nursing practice. Clinical judgement – the practical wisdom that enables us to recognise what matters, weigh competing considerations and act responsibly under uncertainty – remains at the heart of what we do (Kavanagh and Sharpnack, 2021; Manetti, 2019). Yet the environments demanding sound judgement often provide little time or space for it. Multimorbidity proliferates, documentation multiplies, and organisational incentives frequently reward efficiency over reflection. Enter artificial intelligence (AI): a technology promising precision and speed beyond human cognition, from pattern recognition in diagnostics to algorithmic triage and decision support (Gaber et al., 2025; Taylor et al., 2025).

The question we face is not whether AI is powerful, but how it can support rather than undermine the interpretive, relational, and ethical work that defines nursing judgement. Drawing on philosophical insights about practical wisdom, we explore this tension and outline principles for ensuring technology serves our deepest professional commitments.

## Rediscovering practical wisdom

To address this question, we draw on Aristotle’s distinction between *episteme* (scientific knowledge), *techne* (technical skill), and *phronesis* (practical wisdom). *Phronesis* is the capacity to deliberate well about what is good and fitting in particular circumstances – to see what matters, balance competing goods and act responsibly despite uncertainty (Bartlett and Collins, 2011). It cannot be reduced to calculation but develops through repeated engagement with life’s contingencies, guided by virtue and oriented to human flourishing.

Clinical judgement in nursing exemplifies this practical wisdom. It transcends mere evidence application or procedure execution, representing a modern expression of *phronesis* that integrates knowledge, skill, and prudential deliberation towards patient good.

Contemporary nursing frameworks support this view. Tanner’s (2006) model of noticing, interpreting, responding, and reflecting emphasises judgement’s iterative, context-bound nature. Benner’s (1984) developmental account shows how experiential learning refines clinical intuition over time, aligning with Aristotle’s claim that practical wisdom arises through experience with particulars rather than theoretical instruction alone. Adapting Cognitive Continuum Theory, Standing’s (2008) nine modes of practice illustrate how nurses move dynamically between intuitive and analytic processes.

Recent concept analyses reinforce these insights. Connor et al. (2023) defined clinical judgement as reflective reasoning drawing on available data and extensive knowledge, highlighting attributes of clinical reasoning, contextual application of knowledge and reflection alongside consequences including decision-making, reflective practice, patient-centred care and clearer communication. O’Connor et al. (2023) argued that existing decision-making models struggle with present-day complexity and digital technologies, calling for theoretical renewal recognising uncertainty and values-laden trade-offs in contemporary practice.

This convergent picture aligns remarkably with Aristotelian insights: clinical judgement is multifaceted, developmental, and ethical – both cognition and character.

## The paradox of AI

We now stand at the intersection of human wisdom and machine intelligence, where algorithms can detect faint signals in noise, process vast datasets without fatigue, and generate real-time forecasts (Topol, 2019). Properly validated, such tools can extend human perception, foreground relevant variables and counter some well-described biases. On the other hand, the very success of AI invites a mode of practice in which clinicians merely *consult* a device rather than *exercise* their own judgement. Eisikovits and Feldman (2022: 190) warned that by steadily taking over the contexts in which humans practice practical judgement, AI risks ‘*innovating us out of moral competence’*. Their analogy to United States Judge Lois Forer, who resigned when sentencing guidelines eclipsed her capacity to deliberate, is instructive for nursing: even when algorithmic recommendations are accurate on average, the loss of the act of judging undermines moral agency and professional identity. If what matters in clinical judgement is not only the outcome but also the *exercise* of prudence in particular circumstances, then technologies that displace the deliberative work of nurses threaten the very conditions under which *phronesis* is formed.

This danger is not merely theoretical. Aristotle’s account implies that *phronesis* cannot be taught abstractly or imported by protocol; it is acquired through practice in the particular, through repeated cycles of perceiving, weighing and acting, followed by reflection. Educators recognise this in the emphasis on supervised practice, reflective journaling, and narrative debriefing; experienced clinicians recognise it in the tacit, embodied sensibilities that develop over years at the bedside. If AI systems are implemented in ways that short-circuit these formative cycles – for example, by presenting decontextualised ‘answers’ without reasons, or by embedding default pathways that clinicians learn to follow uncritically – then the apprenticeship of judgement is diminished. Over time, even highly skilled practitioners may begin to deskill, not because they forget facts, but because their opportunities to *deliberate well about particulars* have been outsourced.

## Reconciling AI and *phronesis*

At this juncture, a false binary often appears: either we must resist AI to preserve human judgement, or we must defer to AI to secure accuracy and efficiency. Philosophical debate suggests a more constructive path. Tsai and Ku (2025) distinguished three principles for human–AI engagement. According to *epistemic fulfilment*, flourishing requires maximising our exercise of rational capacity; according to *epistemic deference*, we should hand decisions to AI where it is epistemically superior; according to *epistemic heed*, we should continue exercising human judgement to the greatest extent possible while attending carefully to AI’s outputs. The third principle offers a practical ethic for nursing. It does not romanticise human infallibility or demonise computation; rather, it insists that algorithmic recommendations become *inputs* for prudential deliberation, not *substitutes* for it.

What would *epistemic heed* look like in practice? It requires deliberately designing systems that provoke and support, rather than replace, clinical reasoning. On the perceptual side, AI can extend noticing by continuously scanning data and flagging emerging patterns, while making explicit the features that drove an alert so that nurses can evaluate their salience in context (Tanner, 2006). On the analytic side, AI can scaffold interpretation by presenting plausible differentials, projecting trajectories under alternative interventions or surfacing relevant evidence – again with enough transparency to allow critical appraisal (Benner, 1984; Standing, 2008). On the reflective side, AI can help document decision pathways, capture justifications, and facilitate debriefings that feed forward into learning (Twabu, 2025). In each case, the aim is not to bypass cognition but to cultivate it: to make tacit reasoning visible, to anchor attention on what matters and to expand time and cognitive bandwidth for deliberation with patients and colleagues. In Aristotelian terms, AI may contribute to *episteme* by aggregating knowledge and to *techne* by automating tasks, but the prudential synthesis that constitutes *phronesis* remains irreducibly human.

## Education and the apprenticeship of judgement

The developmental literature reinforces this approach. Rogoft (1990) described learning as an *apprenticeship in thinking*, in which novices gain higher-order reasoning through guided participation in sociocultural practice. Nursing education exemplifies this: judgement is learned in the company of others, with mentors who model perception and framing (what to notice), explanation (how to make sense) and enactment (how to respond), and with peers who challenge and refine interpretations. In our view, AI is not merely a calculator; it can also serve as a pedagogical instrument. Twabu (2025) argued that AI can enhance Cognitive Load Theory and multimedia learning frameworks by managing extraneous load, sequencing information to support schema formation, and providing personalised feedback. If routine search, collation, and formatting can be offloaded to machines, and if AI tools can present information at the right grain size and moment, learners can devote scarce cognitive resources to what only they can do: deliberate prudently, articulate values, and integrate patient preferences and contextual constraints into action. The result is not the elimination of judgement but the expansion of its *teachable moments*.

Of course, none of this is automatic. Over-reliance on automation can quietly erode critical thought and promote deskilling (Abdelwanis et al., 2024; Natali et al., 2025; Pavuluri et al., 2024). If algorithms are opaque, nurses may either reject them reflexively or accept them uncritically; neither response supports good care (Amann et al., 2020). Without careful attention to workflow, digital tools produce alert fatigue and distraction rather than clarity (Michels et al., 2025; Shanmugasundaram and Tamilarasu, 2023). Accountability can be displaced from practitioners to systems in ways that undermine professional responsibility. And if equity and ethics are not designed into AI from the start, technologies may amplify existing disparities (Walker et al., 2023; Wei et al., 2025). These risks argue not for retreat but for leadership: nurses must help set requirements for explainability and usability, participate in validation and post-deployment surveillance, and insist that AI adoption be judged not only by predictive metrics but also by its effects on the cultivation of clinical *phronesis*.

## Charting the path forward

There are practical implications for research, education, and policy. Research should move beyond performance metrics to ask whether AI alters the opportunity structure for judgement: does it foster noticing, deliberation and reflection, or diminish them? Mixed-methods studies, including ethnography and cognitive task analysis, can illuminate these effects. In education, curricula should develop not only digital literacy but also philosophical literacy – the capacity to distinguish calculation from prudence and to interrogate algorithmic suggestions in dialogue with patients. Simulation can integrate AI outputs as objects of critique rather than directives to follow, and assessment should reward articulated reasoning rather than alignment with ‘AI-endorsed’ answers. In policy, procurement must prioritise transparency, usability and support for reflective practice, while governance ensures that incentives align with ethical commitments rather than throughput alone.

Seen in this light, ‘from data to meaning’ is a practical programme. Data without judgement are noise; judgement without data is myopic. The promise of AI is not to replace one with the other, but to build sociotechnical systems in which each strengthens the other. When AI surfaces patterns, nurses decide which are meaningful; when it projects consequences, they weigh them against values and context; when it records rationales, teams reflect and learn. Such arrangements preserve what is distinctively human in nursing – interpretation, relational attunement, ethical responsibility – while making disciplined use of what machines do well.

This reframes debates about scope. One view is that AI should be confined to tasks where it has comparative advantage. Stokes and Palmer (2020) argued, from the ethics of caring, that machines must not usurp those aspects of care requiring presence and reciprocity. A complementary criterion is that the exercise of prudential deliberation at the heart of clinical judgement must also remain with nurses. Practical wisdom is not optional; it is the excellence that animates the profession.

None of this denies that AI may outperform human judgement in certain domains, nor does it idealise intuition. What matters is that the ends of nursing – flourishing, justice, and dignity – cannot be reduced to prediction. As Aristotle noted, deliberation is concerned with means, not with ends (Bartlett and Collins, 2011). AI may assist with means, but prudence must still decide which are fitting, for whom, and at what cost. A ‘good’ AI in nursing is thus measured not only by accuracy but also by whether it sustains the conditions in which *phronesis* can be practised and taught.

## Conclusion: Preserving what makes practice human

Nursing practice is sustained by clinical judgement understood as practical wisdom – the integration of knowledge, skill, and ethical discernment in action. AI introduces both risks and opportunities. As a substitute, it risks hollowing out one of our profession’s defining capacities. As a cognitive partner, guided by the principle of epistemic heed, it can enrich perception, deliberation, and reflection while leaving prudence with nurses.

The central task is ensuring AI serves as a partner in the journey from data to meaning. This means designing systems that provoke reflection, scaffolding the apprenticeship of judgement, and maintaining professional responsibility for ethical deliberation. The end of nursing remains the flourishing of persons and communities. AI may assist with means, but the responsibility for determining what is good, just, and fitting belongs to us.

The future of clinical judgement will not be determined by technology alone, but by whether we insist on preserving and cultivating the wisdom that makes our practice distinctly human. In navigating this challenge, we have the opportunity not merely to adapt to technological change, but to shape it in service of our deepest professional commitments.
